# Short term energy consumption forecasting using neural basis expansion analysis for interpretable time series

**DOI:** 10.1038/s41598-022-26499-y

**Published:** 2022-12-29

**Authors:** Abdul Khalique Shaikh, Amril Nazir, Imran Khan, Abdul Salam Shah

**Affiliations:** 1 Department of Information Systems, Sultan Qaboos University, Muscat, Oman; 2 Department of Information Systems and Technology Management, Zayed University, P.O. Box 144534 Abu Dhabi, United Arab Emirates; 3 Department of Computer Science, Sultan Qaboos University, Muscat, Oman; 4 Department of Computer Engineering, University of Kuala Lumpur (UniKl-MIIT), Kuala Lumpur, Malaysia

**Keywords:** Information technology, Computer science, Software, Energy science and technology

## Abstract

Smart grids and smart homes are getting people’s attention in the modern era of smart cities. The advancements of smart technologies and smart grids have created challenges related to energy efficiency and production according to the future demand of clients. Machine learning, specifically neural network-based methods, remained successful in energy consumption prediction, but still, there are gaps due to uncertainty in the data and limitations of the algorithms. Research published in the literature has used small datasets and profiles of primarily single users; therefore, models have difficulties when applied to large datasets with profiles of different customers. Thus, a smart grid environment requires a model that handles consumption data from thousands of customers. The proposed model enhances the newly introduced method of Neural Basis Expansion Analysis for interpretable Time Series (N-BEATS) with a big dataset of energy consumption of 169 customers. Further, to validate the results of the proposed model, a performance comparison has been carried out with the Long Short Term Memory (LSTM), Blocked LSTM, Gated Recurrent Units (GRU), Blocked GRU and Temporal Convolutional Network (TCN). The proposed interpretable model improves the prediction accuracy on the big dataset containing energy consumption profiles of multiple customers. Incorporating covariates into the model improved accuracy by learning past and future energy consumption patterns. Based on a large dataset, the proposed model performed better for daily, weekly, and monthly energy consumption predictions. The forecasting accuracy of the N-BEATS interpretable model for 1-day-ahead energy consumption with “day as covariates” remained better than the 1, 2, 3, and 4-week scenarios.

## Introduction

The concept of smart technologies is gaining popularity in vibrant communities. Smart grids and smart homes are some of the facilities provided by modern smart cities. The smart grids serve as energy production units to provide unstoppable energy to smart homes^1^. The demand for smart home energy emerges in the need for a smart energy consumption prediction mechanism in the smart grids so that production units can produce the required amount of energy per resident’s demand. This concept saves the resources of production and reduces energy wastage^2^. Now researchers are focusing on making smart grids more intelligent to predict the energy consumption of the connected houses and produce energy with less involvement of humans in the energy production process.

The current business models of the grids are more focused on energy production without consideration of future demands and having information about the customers who will be connected with grids due to the rapid construction of new buildings^3,4^. The advancements in smart homes have increased the burden on smart grids; hence energy consumption has also increased^5,6^. Current smart city facilities emphasize automation and security; companies are now focused on making smart homes, smart grids, and smart cities more energy-efficient. The research in smart homes focuses on designing energy-efficient appliances and optimizing energy by devices as per external weather conditions^7,8^. Many aspects of smart homes require automation, including lighting, security, heating, and air conditioning^9^. Besides smart grids, it is also important to improve building energy efficiency. In fact, 70% of businesses focus on making buildings more energy-efficient to reduce energy consumption^10,11^. Various protocols are used in the home and grid network, including Zigbee, KNX, and Z wave^12,13^. Also, the devices can be connected via Wi-Fi or wired networks like Ethernet. Bluetooth can also be utilized in smart homes for short-range communications^11^. Security and energy efficiency are two critical concerns regarding smart homes and smart grid adaptability^14,15^, and are the areas of research where scientists are making progress every day. This paper aims to improve the energy efficiency of the smart grid production unit in accordance with future consumer demands while taking into account energy production costs and wastage.

In the traditional operating environment of smart grids, companies predict the demand for energy for the next day, month, and year. The energy production process can be improved in smart grids, and energy savings can be achieved through prediction methodologies. The prediction allows the customers and production companies to anticipate how much energy will be consumed in the future^16–18^. Researchers have divided load forecasters into three categories, depending on the forecasting horizon. Forecasting horizons of up to one week are defined as short-term load forecast (STLF). A medium-term load forecast (MTLF) predicts load profiles that range from 1 week to 1 year in advance, and a long-term load forecast (LTLF) predicts load profiles from one year into the future^19^. The proposed study focuses on STLF using the dataset acquired from the residential sector. Residential buildings consume more energy than commercial buildings, therefore, to overcome the increasing energy demand in the future, we need a system that saves more and more energy^20^. More energy-saving can be achieved by real-time monitoring energy consumption in smart grids^21^. Traditionally, researchers have used machine learning algorithms and statistical methods to predict energy consumption over the last few decades^22^. Until very recently, prediction models have faced significant challenges due to the nature of data and noise. Deep learning models perform better when pre-processing is performed. However, most methods cannot predict energy consumption due to noise and improper behavior of the energy consumption data^23^. The N-BEATS method has been recently introduced for the time series data and has performed better on benchmark datasets. The proposed model uses N-BEATS, which remains suitable for solving complex patterns of energy consumption data. The interpretability feature of N-BEATS makes this unique model^24^. Because of its complexity and different number of days of every month in the calendar, traditional methods need help dealing with time series data compared to N-BEATS.

The focus of the proposed model is to make the smart grids intelligent enough to behave according to the requirements of future energy consumption of multiple customers^25^. Energy production control and monitoring are the most critical topics related to energy efficiency in smart grids. The model also focuses on the optimal use of smart grid resources. The other main focus of the model is to learn the energy consumption of current customers, use that information to predict the potential energy consumption of new customers.

Several researchers have developed energy-efficient models to deal with smart grids’ energy consumption optimization and prediction issues. Due to a lack of data (for multiple customers), the models cannot predict energy consumption accurately when applied to data of multiple customers^26^. Previous models considered only future energy consumption without considering customer behavior. Adaptability and ease of use are the main disadvantages of traditional methods. This paper aims to improve the prediction accuracy of deep learning algorithms using pre-processing of the data and including the covariates to learn the exact pattern of past and future energy consumption. The proposed model enhances and fine-tunes the newly introduced method of Neural Basis Expansion Analysis for Interpretable Time Series (N-BEATS) with an extensive dataset of the energy consumption of 169 customers. Further, to make sure the validity of the results of the proposed model, a performance comparison has been carried out with the Long Short Term Memory (LSTM), Blocked LSTM, Gated Recurrent Units (GRU), Blocked GRU and Temporal Convolutional Network (TCN) available in the Darts Python library. The model has performed better with the 43 (test data) customers’ daily, weekly, and monthly energy consumption predictions, proving the proposed model’s efficiency on a big dataset. The contributions of this paper are summarized as follows:

- An N-BEATS-based model that considers the behavior of customers (customer-based) is developed for forecasting days, weeks, and months in advance for demand-side management.
- The model considers data having the consumption behavior of multiple customers compared to the traditional methods. Considering the data of multiple customers makes the model unique and reliable for the smart grid.
- The N-BEATS model performs a time-series analysis of the input and the maintenance of time-series behavior as part of the training process.
- A high-dimensional data processing model is developed to simulate the behavior pattern of load consumption over a specific period, which eliminates the problem of over-fitting caused by changes in the data pattern over time due to varying data patterns.
- A variety of state-of-the-art deep learning algorithms, including LSTMs, interpretable LSTMs, GRUs, interpretable GRUs and TCNs, are used to evaluate the proposed N-BEATS model.

The organization of rest of the paper is as follows. The “Related work” section presents the literature review. The “Research design and methods” section discusses research design and methods. The “Results and discussion” section presents the experimental results and detailed discussion; finally, the “Conclusion” section presents the study’s conclusion.

## Related work

Energy consumption forecasting remain a hot topic for researchers; hence different studies have been published in the literature. The focus areas of studies vary from pricing schemes to energy prediction techniques in different domains. Researchers have evaluated how response time and non-linearity impact system identification accuracy in energy forecasting models for buildings. The other technique proposed in Ref.^27^ is classifying buildings into high-power and low-power consumption buildings based on the multi-layer perceptron and random forest^27^. It helps to identify the buildings that consume too much energy and provide them with energy for their needs. In addition to optimizing energy consumption, the classification methods notify customers to change their energy consumption behavior^28^. Initially, the classification methods do not help reduce consumption but only notify the authorities. The optimization frameworks also remain helpful for proper energy distribution. Hui et al.^29^ proposed a real-time local electricity market (LEM) framework to maximize inverter-based HVACs’ regulation potential with multiple DERs, and developed a distribution network optimization framework. Users can use it to evaluate transactive capacity in LEMs to determine regulatory capacities. The LEM also avoids real-time iterations, easing participation difficulties for smaller users. The combination of prediction and optimization algorithms have been used in the smart grid environment for various purposes, including energy management^30–33^. These methods focus on integrating demand, storage and energy production. The adaptive elements and forecasting techniques manage grid resources optimally. Ullah et al.^34^ proposed a hybrid deep learning model to detect electricity thieves in smart grids. Under-sampling, also known as a near miss, solves the class imbalance problem. With AlexNet, the curse of dimensionality issue has been handled, while adaptive boosting (AdaBoost) classified normal consumers and energy thieves. The tuning of hyper-parameters remain critical to achieving better prediction accuracy; hence a bee colony optimization algorithm has been used to tune the AdaBoost, and AlexNet^35^. Comparing the hybrid model to its counterparts, the proposed hybrid model achieves maximum classification accuracy. Han et al.^36^ proposed a novel approach to model smart buildings to assess energy consumption based on the concept of physical-data fusion modeling (PFM). Ye et al.^37^ proposed a theoretical benchmark for optimizing the coordination of local electricity markets (LEM) using a system-centric model. The approach serves as a model-free coordination method for consumer-centric LEM. Authors have used the multi-agent deep reinforcement learning method to integrate multi-actor attention-critic, and prioritized experience replay approaches. The proposed LEM design successfully compresses flexibility services (FS) provision functions and local energy trading functions, remaining more effective than previous methods. The most prominent studies focused on pricing schemes in the smart grid environment. Aurangzeb et al.^38^ developed a fair pricing strategy (FPS) based on power demand predictions using an extreme learning machine (ELM) to save up to 11% of the cost of electricity. Mansouri et al.^39^ propose a novel approach for microgrid scheduling and distribution feeder reconfiguration (DFR) considering load demand, power production and market price. The simulation findings reveal that when the distribution system operator (DSO) can alter the system, the divergence from ideal microgrid scheduling is significantly lower than in cases where the system design is fixed. Wu et al.^40^ present an innovative predictability model that multiple factors and optimization algorithms can interpret. This model performs a variational mode decomposition using a wind speed sequence with several parameters of temporal fusion transformers (TFTs) optimized using adaptive differential evolution. Liu and Wu^41^ used an adjacent nonhomogeneous gray model to predict the consumption of renewable energy in Europe by weighing the latest value compared with the historical data based on the principle of adjacent accumulation. The social media information-based model of oil market forecasting of the US is another dominant forecasting model by Wu et al.^42^.

The forecasting has been carried out in two different areas focusing on smart homes and smart grids. The energy prediction in the smart grid environment remains critical as the grid remains responsible for the power supply and communication with the production units. However, it is necessary to understand and critically evaluate the models of smart homes and grids. The forecasting has been divided into three categories based on the forecasting horizons as; STLF, MTLF and LTLF^19^. The studies focusing on three forecast horizons have been critically evaluated to identify the limitations and research gaps.

### Short-term load forecast (STLF)

Due to the higher production cost of electrical energy, production companies, scientists, and researchers are trying to optimize energy usage and production to avoid wastage and excess energy production. The models considering energy consumption forecasting up to one week are categorized as STLF. Most studies have examined energy consumption predictions hourly, daily, and weekly. The half-hourly energy consumption prediction has been very rare in studies^43,44^. Considering the complexity and cost of the calculation, most of the research concentrates on the hourly and daily predictions of energy. Various algorithms have shown better accuracy, like using a hybrid approach that uses switching delayed particle swarm optimization (SDPSO) for short-term load forecasting; Zeng et al.^45^ used an extreme learning machine and SDPSO algorithm for short-term load forecasting. Predictions are for the short-term, which are mainly based on 1 h to 1 week. With the enhanced capabilities of the SDPSO, a global search can be performed to reach the optimal solution. The SDPSO has been used in extreme learning machines to optimize hidden node parameters. Although the hybrid models improve accuracy, they also increase the complexity of the system^46^. Hence, the model has higher complexity and more calculation time than the single algorithm. The complexity of the model makes it unsuitable for the smart grid environment^47^. A comprehensive study on the short-term energy prediction methods has been published^47^, and it covers the methodological perspectives of the different models. The adaptive method of short-term load forecasting using self-organized maps and SVM by Fan et al.^48^ also contributed to the field of energy efficiency.

Ramos et al.^49^ focused on the energy consumption prediction of a building involving sensors and device consumption recording. They analyzed two prediction methods: k-Nearest Neighbor and artificial neural network (ANN). A multi-armed bandit algorithm is used in the decision-making process in the reinforcement learning framework to establish the most significant possible algorithm in each interval of five minutes, thus enhancing prediction accuracy. Various exploration alternatives have been tested with reinforcement learning in upper confidence bounds, and greedy algorithms^49^.

Torres et al.^50^ used a long short-term memory (LSTM) network to forecast short-term energy consumption due to its capability of dealing with sequences of time series data. Before using a coronavirus optimization algorithm (CVOA)^51^, the best values for various hyper-parameters were obtained by calculating how the SARS-Cov-2 (CVOA) virus spreads. With the optimal LSTM, the electricity demand has been predicted with a 4-h forecast horizon and compared with CVOA. As a comparison, recent deep neural networks have been optimized with grid search techniques, including temporal fusion and deep feed-forward neural networks.

Karijadi and Chou^52^ proposed a hybrid approach using long short-term memory (LSTM) and random forests (RF) to estimate building energy consumption. They transformed energy consumption data into multiple components and predicted the highest frequency component using RF, then LSTM for the remaining components. Jogunola et al.^53^ developed a hybrid deep learning architecture to predict commercial and residential building energy usage accurately. The bidirectional BLSTM designs, convolutional neural networks (CNNs), and auto-encoders (AEs) with bidirectional long short-term memory (LSTM)^54^. The AE-BLSTM and LSTM layers make predictions, while the CNN layer gathers features from the dataset. The findings improved calculation time and mean squared error compared to a vanilla LSTM and CNN BLSTM-based framework (EECP-CBL). Fu et al’s^55^ models performance often improve with increased computation time when using deep reinforcement learning (DRL) for energy usage estimation. The deep-forest-based DQN (DF-DQN) proved more accurate than the deep deterministic policy gradient (DDPG).

Bilgili et al.^56^ used long short-term memory (LSTM) neural network, adaptive neuro-fuzzy inference system (ANFIS) with subtractive clustering, ANFIS with fuzzy c means, and ANFIS with grid partition for the short-term one-day ahead energy consumption prediction. All of the ANFIS models were surpassed by the LSTM model. Peng et al.^57^ used wavelet transform and LSTM to predict energy consumption accurately. Somu et al.’s^58^ model used LSTM and kCNN for energy consumption forecasting because of the spatiotemporal dependencies in the energy consumption data.

### Medium-term load forecast (MTLF)

The MTLF forecasting models range from one week to one year. As a result of the difficulty in finding large datasets, the previous studies have mainly looked at weekly rather than one-year forecasting^59,60^. On the other hand, deep learning methods require larger datasets for proper training, but only some researchers have succeeded in improving accuracy with small datasets. The 1-week to 1-month MTLF method by Fayaz and Kim^61^ has used a deep extreme learning machine model to predict energy consumption in smart homes and compared it with the adaptive neuro-fuzzy inference system (ANFIS) and an artificial neural network (ANN). Deep extreme learning outperformed the other two algorithms, using the method of trial and error for activation functions and the selection of hidden layers. The disadvantage of the trial and error method is the extra calculations to find the optimal solution^62^. The problem with the small datasets is that when time series is used, it reduces the model’s performance because of the limited number of data^63^. The time series prediction requires sufficient data so that deep learning algorithms can learn the data patterns for the prediction. The other most prominent deep learning-based MTLF techniques are^64–66^, although they fall under the STLF as well because most of the authors have considered STLF and MTLF in their studies^67,68^. The quantile regression and statistical methods also performed better for the MTLF^69–71^. Wahid et al.^72^ used the multi-layer perceptron, logistic regression and random forest techniques to predict daily energy consumption. However, it has limitations, as the authors have used a small dataset. Because the statistical methods are simple, the algorithms perform poorly when data from multiple customers is incorporated^73^. In comparing the three classifiers, logistic regression was better than the other two methods^72^. The deep learning methods have been applied to the distribution feeders for load forecasting^74^. Jogunola et al.^75^ assessed energy usage in commercial buildings in a post-COVID-19 environment while investigating the influence of digitization to uncover potential new opportunities using actual power consumption data. The primary goal was to determine how energy demand varies with occupancy rate. The findings show that the reduction in energy demand is different from occupancy, resulting in high energy costs. Because inefficient energy use increases consumption, improving energy efficiency techniques such as time of use and scheduled energy use can help conserve energy.

### Long-term load forecast (LTLF)

The long-term load forecasting techniques have been presented using the machine learning and statistical methods. The particle swarm optimization performed better for the LTLF model in the Kuwait energy demand network^76^. The problem with the LTLF is the requirement of big datasets so that models can be trained, although the statistical methods can perform better with the small datasets compared with the deep learning models^77–79^. The alternative to the big data has been considered as monthly energy prediction for 1 year, instead of considering yearly energy consumption datasets^80,81^. The backprogation-based methods have performed better with the LTLF as the adjustment factor enhanced the performance of traditional BPA^82^. The LTLF has been carried out using different optimization algorithms for the electricity’s load of the Sivas province of Turkey^83^. The model helps to meet the energy demand of the province. The literature review has revealed that only a few authors have considered the LTLF due to the unavailability and complexity of the data. The studies mainly considered MTLF for a longer duration of months and extended it up to one year, so the LTLF and MTLF remained interlinked with one other. The other significant problem with the LTLF models is the consideration of data from very few customers; hence these models need to be tested on larger datasets so that they can be implemented in the smart grid environment. Due to the short duration of the data, many authors have yet to consider the time series data, which is very important while predicting the energy consumption load.

A detailed review of these methods has revealed that the deep learning models remain successful in predicting short and long-term energy consumption. For the shorter datasets, the statistical models have performed better. The main issue with these models is to deal with the complex sequences of the time series data hence enhancing the need for a robust model to tackle the issue of energy consumption prediction and prediction of energy consumption behavior of new customers who are going to join the smart grid in future. The majority of the methods are focused on complete datasets without consideration of the individual energy consumption behavior of the customer. Hence, the model needs to handle the time series data better and handle multiple customers’ complex energy consumption behavior.

## Research design and methods

The proposed methodology of future energy consumption prediction aims to enhance the accuracy of the N-BEATS interpretable algorithm^84^. The methodology starts with pre-processing of the data and removal of the outliers. The second step is smoothing data and then an N-BEATS interpretable model based on the *n* number of stacks containing *n* number of blocks with fully connected layers (FC) stacks having ReLU as activation function with backcast and forecast functions. The proposed model can be seen in Fig. 3.

For the determination of the optimal structure of N-BEATS interpretable, a trial and error method has been adopted. Finally, a model has been designed with optimal structure with an input chunk size of 30, an output chunk size of 15, 10 block size, 20 hidden layers having layer width of 512, a learning rate of 1e−3, number of epochs from 100–200 having epoch validation period of 1, and considering the batch size of 1000–1500. The model uses ReLU as an activation function for the hidden layers. The parameter setting of the other algorithms can be seen in Table 1. The methods used in this study were carried out by relevant guidelines and regulations.

**Table 1:** Parameter settings of algorithms.

Name of algorithm						
Parameter	LSTM	Blocked LSTM	GRU	Blocked GRU	CN (temporal)	N-BEATS (interpretable)
Activation function	ReLU		ReLU		ReLU	ReLU
Hidden dim/size/widths	20	10	20	10		512
Number of layers	1	1		3	6	20
Random state	0–30	42	0–30	0–42	0	
Training length/input chunk length	30	30	30	30	30	30
Output chunk length	15	15	15	15	15	15
Nr epochs val period		1		1		
Batch size	800–1500	32	200–1500	1000	512	1000–1500
Optimizer	Adam		Adam			
Learning rate	1–3	1–3	1–3	1–3	1–3	
Epochs	200–300	150–250	200–300	150–250	200–250	100–220
Dilation base					2	
Kernel size					5	
Number of filters					3	
Dropout rate	0–0.2	0.1	0–0.2	0.2	0.1	
Number of blocks						10
Future/past covariates	Day series	Day series	Day series	Day series	Day series	Day series

The description of each component of the model can be seen in subsequent sub-sections.

### Database acquisition and description and availability

The energy consumption dataset of 5567 London households has been acquired as it is freely available for non-commercial and research purposes^85^. The duration of the data is from November 2011 to February 2014. The half-hourly data have been converted to daily energy consumption in kWh to reduce the number of readings. The unique id of the customer serves as an identifier for each customer’s energy consumption in the dataset containing a total of 167 million rows. The dataset contains two types of customers, wherein for the experimentation in the proposed study, we have considered the first 169 customers in the dataset having energy prices as per the dynamic time of use (dToU).

The remaining data of customers have been dropped to avoid memory and extensive computation-related issues. Few customers have very high energy consumption compared to the remaining hence have been dropped for further consideration in the experimentation. The dataset has approximately 29 months of energy consumption for each customer. The ratio of 75% for training and 25% for testing has been adopted for experimentation. The data’s insights, like count and some statistical information, can be seen in Table  2.

**Table 2:** Insights of the data set.

Total customers			Customers in training data		Customers in test data		
169			126 (75% of 169)		43 (25% of 169)		

Count	Mean	Std	Min	25%	50%	75%	Max
115584	8.92	6.26	0.0	4.77	7.28	11.46	84.15

### Pre-processing

The dataset contains outliers with sudden energy consumption peaks, making it difficult for the algorithms to forecast future energy consumption. The moving average method has been adopted to remove the possible outliers from the initial dataset. The data have been normalized to a scale of 0–1 using (1), while de-normalization has been achieved using (2).

1 $$\begin{aligned} Normalized (a)= & {} \frac{x(a)-\min (a)}{\max (a)-\min (a)}, \end{aligned}$$

2 $$\begin{aligned} Denormalized (a)= & {} Normalized(a)* (\max (a)-\min (a))+\min (a), \end{aligned}$$

where normalized data *a* have been represented by *Normalized*(*a*). The value being normalized is represented by *x*(*a*). While the *min*(*a*) and *max*(*a*) denote the minimum and maximum values of the dataset.

### Identification and removal of outliers

**Figure 1:**
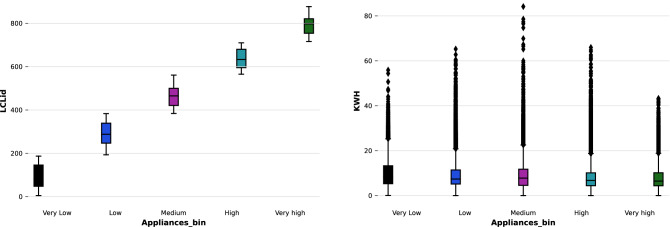
Original data.

When outliers in a dataset are ignored due to errors of omission or because they deviate from the normal statistical distribution in a dataset, machine learning and deep learning algorithms are severely impacted as seen in Fig. 1.

**Figure 2:**
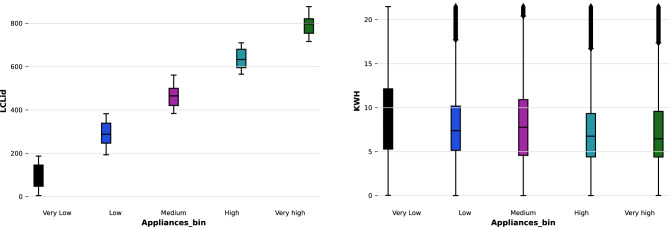
Pre-processed data.

Interquartile Range (IQR) refers to the difference between a dataset’s fourth and third percentiles (the upper and lower quartiles). Therefore, the interquartile range of the dataset would follow a breakup point of 25%. IQRs are used to identify outliers in box plots when expressed as deviations. An outlier is an observation that falls below or exceeds *Q*1 + 1.5 IQR. In the proposed model, the outlier identification and removal in Python have been done using NumPy. The pre-processed data can be seen in Fig. 2. The IQR can be calculated by (3).

3 $$\begin{aligned} OQR=Q3-Q1, \end{aligned}$$

where the upper quartile can be denoted by *Q*3 and lower quartile as *Q*1.

### N-BEATS: neural basis expansion analysis for interpretable time series

**Figure 3:**
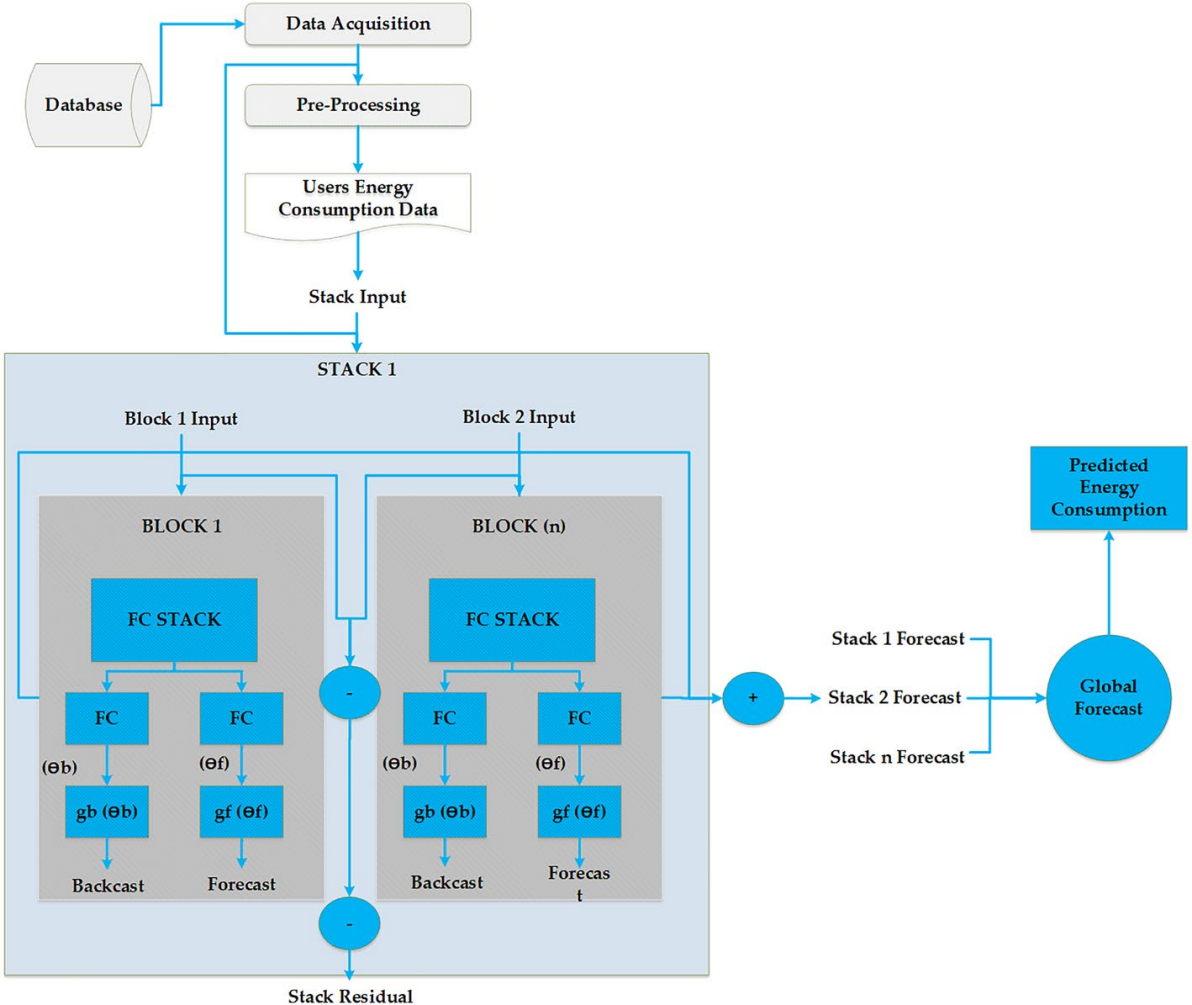
Proposed N-BEATS interpretable model.

The proposed N-BEATS interpretable model can be seen in Fig. 3. It must be highlighted that the functioning, details, idea of the model diagram, functional components and equations of N-BEATS have been taken from the^84^. The reader may refer^84^ for further details of the N-BEATS interpretable algorithm. The major building block of the N-BEATS is the blocks; hence the proposed N-BEATS interpretable contains 10 blocks. For simplicity, Fig. 3 depicts two blocks only. The stacks are responsible for holding different blocks inside; hence Fig. 3 shows 1 stack having 2 blocks. The basic function of $i^{th}$ block is to take input to suppose $a_{i}$ and provide the output of $bx_{i}$ and $by_{i}$. The first block of the N-BEATS interpretable takes input $x_{i}$ along with the look-back windows. In comparison, the last measured observation by the block remains the ending point of the look-back window. The proposed method contains blocks having multi-layers forming a fully connected layers (FC) network with ReLU function. While there is a total of 20 hidden layers having 512 layers width, making a complex deep network. The layers predict the expansion coefficients for forecast $\theta ^ {f}$ and backcast $\theta ^ {b}$ of energy consumption^84^.

The doubly residual stacking has been used to connect all 10 blocks of the proposed model having $g_{b}$ and $g_{f}$ shared among different layers of stacks for the hierarchical aggregation of the forecast. The hierarchical aggregation allows the designing of deep neural networks having interpretable outputs. The forecast horizons *H*, of 1, 7, 14, 21, and 30 have been considered to learn the behavior of energy consumption by different customers. The residual outputs of previous blocks are the input $x_{l}$^84^. The model contains a fully connected network of prediction components with a forecast $\theta ^ {f}_{l}$ and backcast $\theta ^ {f}_{l}$. Every block has output forecast $\hat{y}_{l}$ and backcast $\hat{x}_{l}$, having a length of *H*. The second prediction components with backcast $g^ {b}_{l}$ and $g^ {f}_{l}$ to accept the forecast $\theta ^ {f}_{l}$ and backcast $\theta ^ {b}_{l}$ expansion coefficients. It helps in the production of backcast $\hat{x}_{l}$ and forecast outputs $\hat{y}_{l}$. The block of N-BEATS $l-th$ can be described by Eqs. (4a), (4b), (4c), (4d), (4e) and (4f)^84^.

4a $$\begin{aligned} h_{l,1}= & {} FC_{l,1}(x_{l}), \end{aligned}$$

4b $$\begin{aligned} h_{l,2}= & {} FC_{l,2}(x_{l}), \end{aligned}$$

4c $$\begin{aligned} h_{l,3}= & {} FC_{l,3}(x_{l}), \end{aligned}$$

4d $$\begin{aligned} h_{l,4}= & {} FC_{l,4}(x_{l}), \end{aligned}$$

4e $$\begin{aligned} \theta ^ {b}_{l}= & {} Linear^{b}_{l}(h_{l,4}),\end{aligned}$$

4f $$\begin{aligned} \theta ^ {f}_{l}= & {} Linear^{f}_{l}(h_{l,4}). \end{aligned}$$

The linear layer represents the projection layer as in (5a) while the fully connected layer $FC_{l,1}$ with RelU can be represented by (5b).

5a $$\begin{aligned} \theta ^ {f}_{l}= & {} W^{f}_{l}(h_{l,4}),\end{aligned}$$

5b $$\begin{aligned} h_{l,1}= & {} ReLU (W_{l,1}x_{l}+ b{l,1}). \end{aligned}$$

The purpose of this part is prediction of forecast $\theta ^{f}_{l}$ and backcast $\theta ^{g}_{l}$ coefficients to optimize the accuracy of $\hat{y}_{l}$ by mixture of vector $g^{f}_{l}$.The backcast coefficient uses $g^b_{l}$ for the estimation of $x_{l}$. The functionality of the mapping of expansion coefficients $\theta ^{f}_{l}$ and $\theta ^{b}_{l}$ to output can be denoted by (6a) and (6b).

6a $$\begin{aligned} \hat{y}_{l}&= \sum ^ {dim(\theta ^{f}_{l})} _{i=1} \theta ^{f}_{l,i}v^{f}_{i},\end{aligned}$$

6b $$\begin{aligned} \hat{x}_{l}&= \sum ^ {dim(\theta ^{b}_{l})} _{i=1} \theta ^{b}_{l,i}v^{b}_{i}, \end{aligned}$$

where the forecast and backcast vectors are represented by $v^{f}_{i}$ and $v^{b}_{i}$. While the $i^{th}$ element is denoted by $\theta ^{f}_{l,i}$.

The proposed model uses the N-BEATS interpretable trend model, using the constraints $g^{b}_{s,l}$ and $g^{f}_{s,l}$ same can be determined by (7).

7 $$\begin{aligned} \hat{y}_{s,l}= \sum ^ {p}_{i=0} \theta ^{f}_{s,l,i}t^{i}. \end{aligned}$$

The *t* denoted time vector ranging 0 to $(H-1)/H$, *H* ahead forecasting steps. The detailed calculations and functioning of the N-BEATS interpretable model have been provided in^84^.

### Performance evaluation

The performance of the LSTM, GRU, Blocked LSTM, Blocked GRU, TCN, and N-BEATS interpretable models has been measured using mean absolute error (MAE), root mean square error (RMSE), mean absolute percentage error (MAPE), and the mean square error (MSE)^86^. The reason for selecting these parameters is a consideration in the literature for regression accuracy. Although the MAPE values in the results are higher, the difference between predicted and actual values is minimal; hence even the 1 value further gives a higher MAPE error rate. These performance parameters can be mathematically defined as (8), (9) (10), and (11).

8 $$\begin{aligned} MAE= & {} \frac{1}{N} \sum ^n _{i=1}|A_i-P_i |, \end{aligned}$$

9 $$\begin{aligned} MAPE= & {} \frac{1}{N} \sum ^n _{i=1}\frac{|A_i-P_i |}{A_i} \times 100, \end{aligned}$$

10 $$\begin{aligned} MSE= & {} \frac{1}{N} \sum ^n _{k=0}(A-P_i)^2, \end{aligned}$$

11 $$\begin{aligned} RMSE= & {} \sqrt{\frac{1}{N} \sum ^n _{k=0}(A-P_i)^2}, \end{aligned}$$

where *N* represents total observations, *A* denoted actual and *P* as predicted values.

## Results and discussion

The analysis of achieved results with different deep learning models has revealed that the models have performed better despite some fluctuations in the data and improper energy consumption due to different customers’ energy consumption data. Various scenarios have been considered to evaluate the models and train them accordingly. The scenarios include the energy consumption of 126 training and 43 testing customers for the next day, week, two weeks, three weeks, and four weeks. The main focus of the proposed model is on the performance improvement and design of an interpretable N-BEATS method using proper parameters tuning and the addition of pre-processing steps in the model to smooth the data for better evaluation and adequate training of the model.

### Back-testing

**Figure 4:**
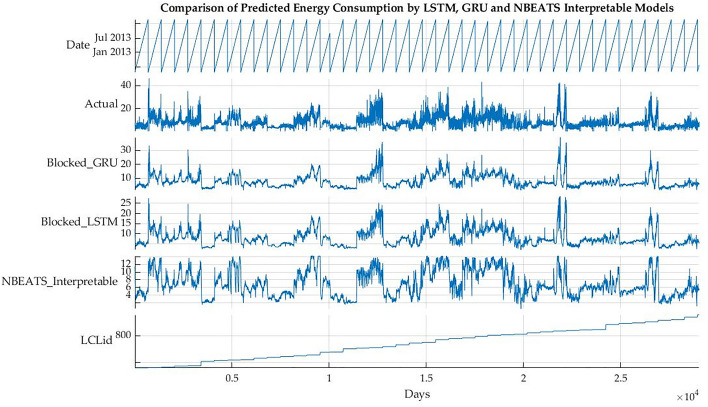
Performance comparison of interpretable models.

The back-testing has been done with different algorithms to evaluate the performance of the proposed model. The testing scenarios for 1, 7, 14, 21 and 30 days ahead have been used. The performance of the 1 day ahead scenario of the interpretable GRU, LSTM and N-BEATS have been graphically represented in Fig. 4. The terms day ahead shows how much energy will be consumed on the following day; similarly, the 7-day and 30-day ahead energy consumption represents how much energy will be consumed on the 7th and 30th days. This strategy helps to understand the performance of algorithms. Most algorithms have performed better regarding the day, week and month ahead energy forecasting. The graphs of the rest of the scenario seem to be similar as the difference between actual and predicted energy consumption remains small; hence it has been decided to only represent the 1 day ahead graphs. The performance of the proposed N-BEATS interpretable model has been compared with the Blocked GRU and Blocked LSTM. The LCLids represent the unique number of customers, while the actual represents actual energy consumption compared with the predicted energy consumption by each deep learning algorithm. The total number of days on the *x*-axis is 29042. The *x*-axis of the graph contains the time (days) duration of energy consumption, while the *y*-axis of figures contains power consumption in kWh. All these models use interpretable LSTM, GRU and N-BEATS versions of the algorithms. The performance of all the other scenarios has been discussed in Table 2.

**Figure 5:**
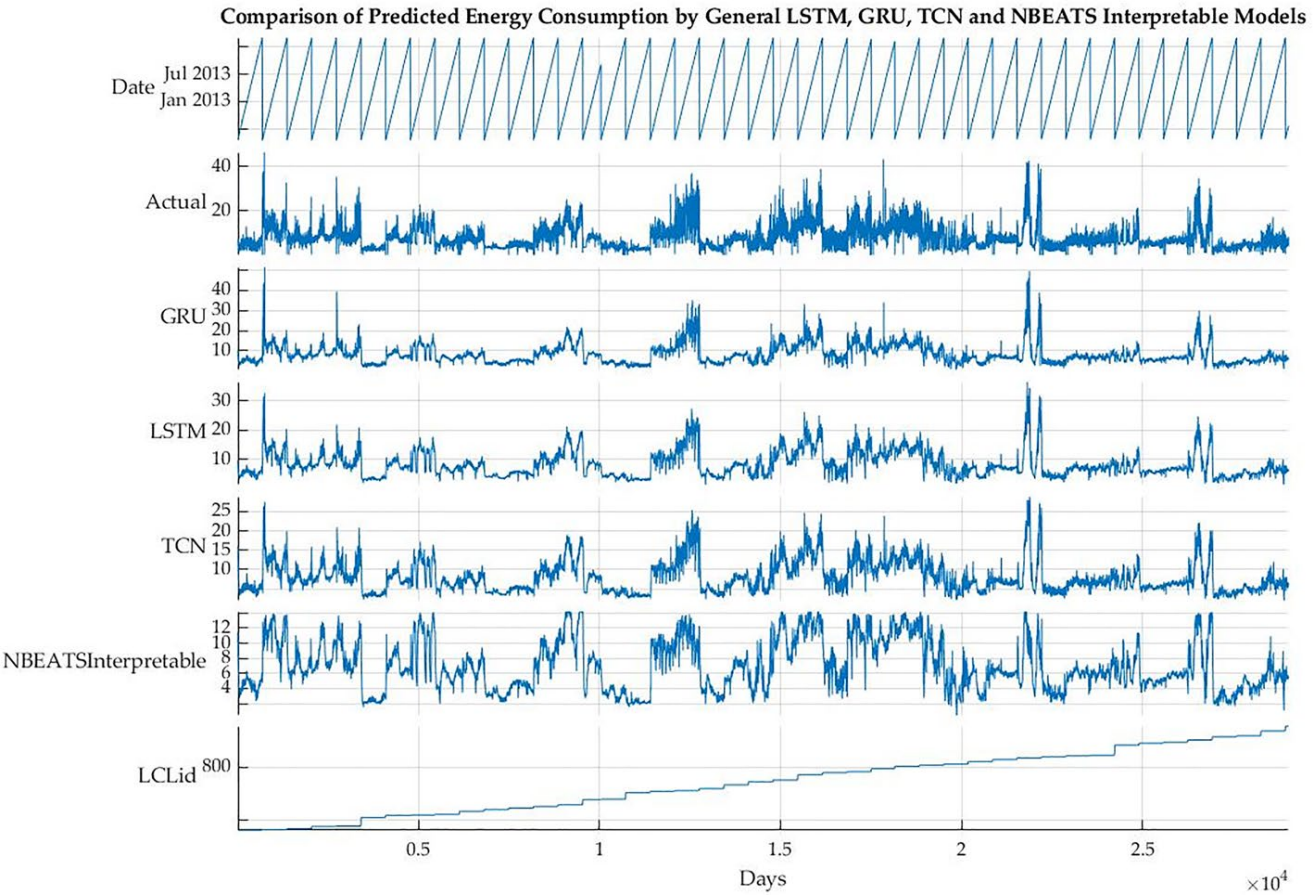
Performance comparison of general models.

In the back-testing, the energy consumption of 43 customers having energy consumption of 29042 days has been used to evaluate the performance of general and interpretable models. It can be noticed from the graph in Fig. 4 that the proposed N-BEATS interpretable model has performed better compared to the LSTM and GRU interpretable models. Figure 5 compared 1 day ahead actual energy consumption and predicted energy consumption by the LSTM, GRU and TCN general models. The performance of the proposed N-BEATS interpretable model has been compared with the general GRU, general LSTM and TCN models. The total number of days on the *x*-axis is 29042. The *x*-axis of the graph contains the time (days) duration of energy consumption, while the *y*-axis of the figures contains power consumption in kWh. All these models use general models except N-BEATS interpretable. It can be noticed from Fig. 5 that the actual energy consumption has fluctuations due to the data of different customers showing different behaviors of energy consumption. Hence the energy consumption pattern remained a challenge for the N-BEATS interpretable algorithm to predict the energy consumption. Even though the proposed model has used normalized data for the training of models and at the time of calculation of statistical parameters (RMSE, MAPE, MSE and MAE), the results have been de-normalized. So the normalization has significantly improved the performance of models despite frequent fluctuations in customers’ energy consumption data. While in the scenario of general models, the performance of N-BEATS interpretable without smoothed data remained lower. The reason for this is the addition of complexity due to ”day as covariates,” making it difficult for the algorithm to learn the complex patterns and different behaviors of the energy consumption by different customers compared to the other general models. Further interpretable models look for every detail of data, affecting the performance compared to general models.

### Performance evaluation criteria

**Figure 6:**
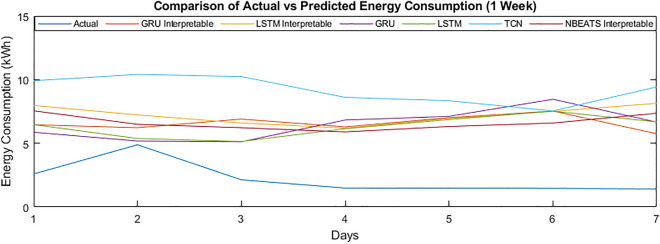
Comparison of actual vs predicted energy consumption (1 week).

**Figure 7:**
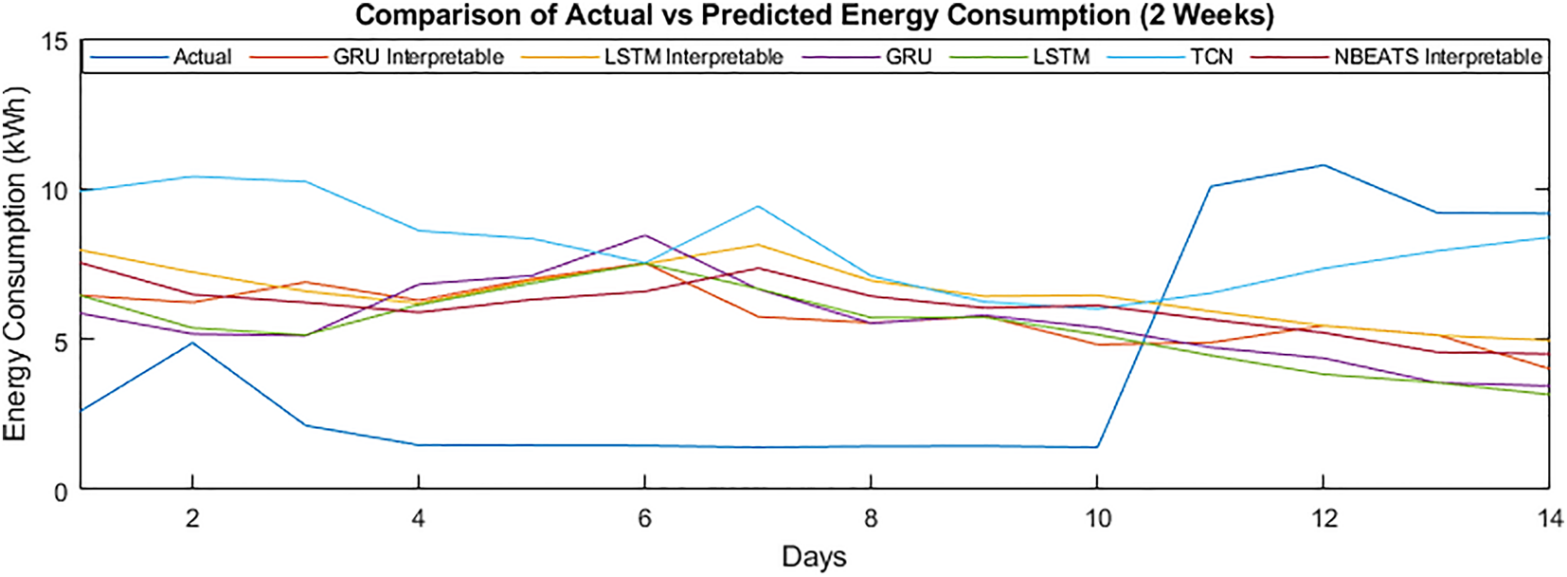
Comparison of actual vs predicted energy consumption (2 weeks).

**Figure 8:**
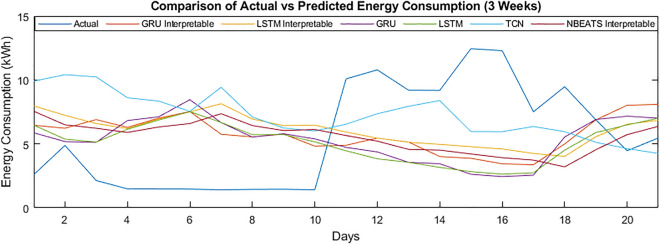
Comparison of actual vs predicted energy consumption (3 weeks).

**Figure 9:**
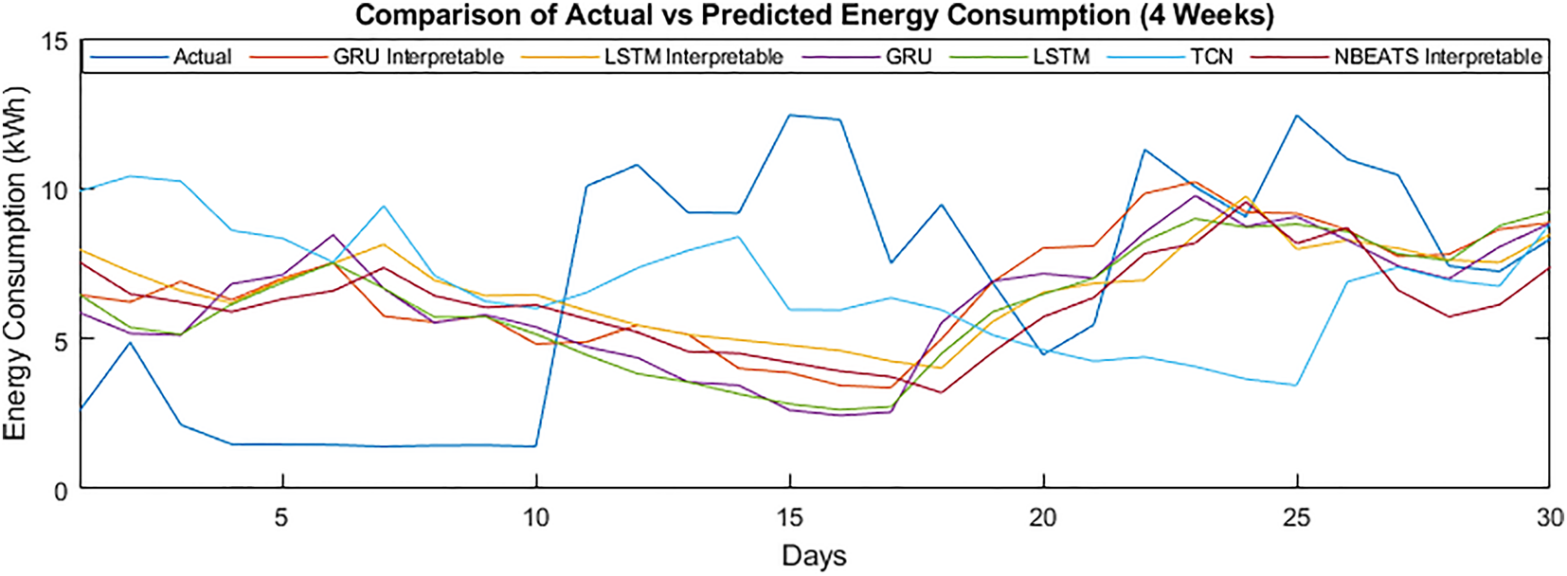
Comparison of actual vs predicted energy consumption (4 weeks).

**Figure 10:**
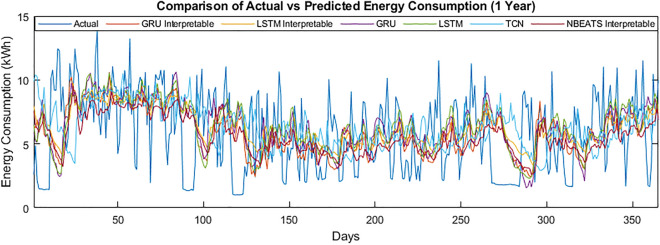
Comparison of actual vs predicted energy consumption (1 year).

Table  3 represents the performance evaluation of all models in the MAPE, MAE, RMSE, and MSE. It can be noticed that the performance of the N-BEATS interpretable has improved with the addition of the smoothing data module. The experimental setup considers different scenarios like the day ahead, 1, 2, 3, and 4 weeks ahead of energy consumption forecasting. For simplicity, the analysis of the day ahead, week, and four weeks ahead have been carried out. However, the full results of all scenarios are provided for self-comparison. The reason for selection is that the energy production decisions are mainly based on day, week, and month ahead energy consumption forecasting. Further, for the graphical representation, we have randomly selected the data for the year 2013 of the customer having ID 789 for the 1, 2, 3 and 4 weeks as Figs. 6,7, 8 and 9. While the energy consumption for the entire year 2013 has been represented in Fig. 10.

**Table 3:** Performance comparison of the deep learning models.

	Prediction model														
Metric	For.Hor.	N Cov.	D. Cov.	N Cov.	D. Cov.	N Cov.	D. Cov.	N Cov.	D. Cov.	N Cov.	D. Cov.	N Cov.	D. Cov.	N Cov.	D. Cov.
		LSTM		Blocked LSTM		GRU		Blocked GRU		CN (temporal)		N-BEATS (Inter.)		N-BEATS (Inter.) Smoothed data	
MAPE	1 day	49.50	46.94	48.78	52.13	47.48	46.14	49.72	58.65	61.68	63.35	55.91	48.57	47.04	48.81
MAE		1.54	1.56	1.53	1.61	1.51	1.63	1.55	1.84	1.99	2.00	1.67	1.73	1.52	1.75
MSE		5.72	6.33	5.81	6.43	5.72	6.49	5.85	9.18	10.05	10.03	6.56	7.23	5.14	6.49
RMSE		2.39	2.52	2.41	2.54	2.39	2.55	2.42	3.03	3.17	3.17	2.56	2.69	2.27	2.55
MAPE	7 days	63.10	55.50	60.50	60.58	56.76	53.20	58.30	61.28	68.61	63.55	59.76	52.99	50.69	51.09
MAE		1.87	1.83	1.80	1.85	1.76	2.08	1.78	1.86	2.08	2.00	1.86	1.98	1.68	1.75
MSE		8.54	8.93	8.15	8.99	8.26	9.95	8.07	9.03	10.06	9.93	8.96	9.28	6.32	6.53
RMSE		2.92	2.99	2.85	3.00	2.87	3.15	2.84	3.01	3.17	3.15	2.99	3.05	2.51	2.56
MAPE	14 days	69.75	59.24	60.84	61.79	60.11	58.83	63.71	65.58	68.39	63.32	63.89	61.16	55.02	53.55
MAE		2.06	2.00	1.90	1.98	1.92	2.47	1.94	2.03	2.07	1.99	2.02	1.94	1.79	1.98
MSE		10.22	10.43	9.38	10.52	9.82	12.83	9.54	10.72	10.02	9.91	10.80	9.63	7.08	8.07
RMSE		3.20	3.23	3.06	3.24	3.13	3.58	3.09	3.27	3.16	3.15	3.29	3.10	2.66	2.84
MAPE	**21 days**	74.83	60.24	63.90	59.47	62.69	62.06	65.86	60.58	76.58	66.59	58.30	63.68	56.24	54.16
MAE		2.21	2.13	2.01	2.38	2.02	2.73	2.05	2.22	2.29	2.09	2.20	2.04	1.88	2.11
MSE		11.60	11.64	10.45	12.66	11.00	15..04	10.64	11.99	11.29	10.97	11.42	10.72	7.66	8.99
RMSE		3.41	3.41	3.23	3.56	3.32	3.88	3.26	3.46	3.36	3.31	3.38	3.27	2.77	3.00
MAPE	**30 Days**	78.71	63.31	66.92	68.65	65.26	64.87	69.49	61.53	78.27	68.01	60.04	66.46	58.79	55.49
MAE		2.35	2.27	2.11	2.18	2.12	2.81	2.15	2.35	2.37	2.19	2.31	2.14	1.95	2.25
MSE		12.88	12.76	11.39	12.07	12.03	16.34	11.56	13.07	12.08	11.92	12.57	11.66	8.22	9.96
RMSE		3.59	3.57	3.38	3.47	3.47	4.04	3.40	3.61	3.48	3.45	3.54	3.41	2.87	3.16

For. Hor. represents the forecast horizon, N Cov. represents no covariates, while the D. Cov. denoted day as covariates. While the Inter. represents the interpretable.

Significant values are in bold.

#### Day-ahead energy forecasting

If we compare the results of N-BEATS interpretable without smoothed data and after smoothed data, significant improvement is shown. The improvement is significant in all performance parameters, for example MAPE has been enhanced from 55.91 to 47.04 in the scenario of “no covariates.” However, in the case with the “day as covariates,” the performance in terms of MAPE has slightly reduced from 48.57 to 48.81. The MAE, has been improved from 1.67 to 1.52 for the “no covariates,” but with the “day as covariates,” the MAE with smoothed data has increased to 1.75 compared to the 1.73 for the original data. The MSE with “no covariates” has been noticed as 6.56 and improved to 5.14. A significant improvement in the MSE of “day as covariates” has been noticed, from 7.23 to 6.49. Similarly, the RMSE with “no covariates” has been improved from 2.56 to 2.27. An improvement in the ”day as covariates” RMSE can be noticed as it has been enhanced from 2.69 to 2.55.

If we compare the performance of the proposed model with the interpretable model Blocked GRU, it can be seen that the proposed model has performed better than the competent model. The MAPE, MAE, MSE, and RMSE of the Blocked GRU with “no covariates” scenario remained as 49.72, 1.55, 5.85, and 2.42. With the “day as covariates,” it has remained at 58.65, 1.84, 9.18, and 3.03.

The interpretable Blocked LSTM has performed slightly better than the Blocked GRU. The MAPE, MAE, MSE, and RMSE of the Blocked LSTM with “no covariates” scenario remained as 48.78, 1.53, 5.81, and 2.41. With the “day as covariates,” it has remained as 52.13, 1.61, 6.43, and 2.54. The addition of the pre-processing module and fine-tuning of the N-BEATS interpretable model with smoothed data has outperformed Blocked GRU and Blocked LSTM in both “day as covariates” and “no covariates” scenarios for the day ahead energy consumption forecasting.

If we compare the results with LSTM general model, the MAPE, MAE, MSE and RMSE, with “no covariates” have remained at 49.50, 1.54, 5.72 and 2.39. These results are slightly better than the N-BEATS interpretable without smoothing and the “no covariates” scenario. On the other hand, the MAPE, MMAE, MSE and RMSE for the “day as covariates” remained at 46.94, 1.56, 6.33 and 2.52, respectively. Comparing these results with the “day as covariates” scenario of N-BEATS interpretable without smoothed data, the LSTM general model has performed better than the N-BEATS interpretable model. Compared with the N-BEATS interpretable with smoothed data and LSTM general model, the performance gap has been reduced, like MAPE, MAE, MSE, and RMSE to 47.04, 1.52, 5.14 and 2.27 in the scenario of “no covariates”and 48.81, 1.75,6.49 and 2.55 for the “day as covariates.” There is a minimum difference between the performance of the LSTM general and N-BEATS interpretable model with smoothed data.

The results of the GRU general model have been analyzed in terms of the MAPE, MAE, MSE and RMSE, with “no covariates”; they have remained as 47.48, 1.51, 5.72 and 2.39 comparatively better than the N-BEATS interpretable without smoothing and the “no covariates” scenario. On the other hand, the MAPE, MAE, MSE and RMSE for the “day as covariates” remained at 46.14, 1.63, 6.49 and 2.55, respectively. Comparing these results with the “day as covariates” scenario of N-BEATS, the GRU general model has performed better than the N-BEATS interpretable model. Compared with the N-BEATS interpretable with smoothed data and GRU general model, the performance of the N-BEATS interpretable model in both scenarios of “no covariates” and “day as covariates” remained better. There is a minimum difference between the performance of the GRU general and N-BEATS interpretable model with smoothed data.

The MAPE, MAE, MSE and RMSE of Temporal Convolutional Network (TCN) compared with “no covariates”; have remained as 61.68, 1.99, 10.05 and 2.17. In this scenario, the N-BEATS has outperformed TCN without smoothing and with smoothing data. The MAPE, MAE, MSE and RMSE for the “day as covariates” remained 63.35, 2.00, 10.03 and 3.17, respectively. Compared with the N-BEATS interpretable with smoothed data and TCN general model, the performance of the N-BEATS interpretable model in both scenarios of “no covariates” and “day as covariates” remained better. There is a minimum difference between the performance of the TCN and N-BEATS interpretable model with smoothed data.

Figure 6 shows the specific results of randomly selected data of customer 789 for the first week of 2013. It can be seen that the data had different patterns for each customer, hence making it difficult for the algorithms to predict the energy consumption in the same range as compared to the actual energy consumption.

#### Week-ahead energy forecasting

It can be observed from the week ahead energy consumption forecasting scenario that the model’s performance has reduced compared to the day ahead scenario. Still, the proposed model has improved the performance in MAPE enhancement from 59.76 to 50.69 in the scenario of “no covariates.” While considering “day as covariates,” the performance in terms of MAPE has slightly improved from 52.99 to 51.09. Although the improvement is minor, model has managed to improve the performance compared to the day-ahead scenario. The MAE has been enhanced from 1.86 to 1.68 for the “no covariates,” and with the “day as covariates,” the MAE remained at 1.75 compared to 1.98. The model has achieved MSE with “no covariates” as 8.96, which has improved to 6.32. The MSE of “day as covariates” has been enhanced from 9.28 to 6.53. Similarly, the RMSE with “no covariates” has been improved from 2.99 to 2.51. An improvement in the “day as covariates” RMSE can be noticed, enhancing it from 3.05 to 2.56.

If we compare the performance of the proposed model with the interpretable Blocked GRU, the MAPE, MAE, MSE, and RMSE with “no covariates” scenario remained at 58.30, 1.78, 8.07, and 2.84. With the “day as covariates,” it has remained at 61.28, 1.86, 9.03, and 3.01.

The interpretable Blocked LSTM performance remained identical to the Blocked GRU. The MAPE, MAE, MSE, and RMSE of the Blocked LSTM with “no covariates” scenario remained at 60.50, 1.80, 8.15, and 2.85. The “day as covariates” has remained at 60.58, 1.85, 8.99, and 3.00. The N-BEATS interpretable model without and with smoothed data has outperformed the Blocked GRU and Blocked LSTM in both “day as covariates” and “no covariates” scenarios for 7 days ahead energy consumption prediction.

The LSTM general model, MAPE, MAE, MSE and RMSE, with “no covariate”, have remained at 63.10, 1.87, 8.54 and 2.92. The “day as covariates” remained at 55.50, 1.83, 8.93 and 2.99. The N-BEATS interpretable with and without smoothing scenarios has outperformed the LSTM general model. If we compare these results with the “day as covariates” scenario of the N-BEATS interpretable without smoothed data, the LSTM general model failed to improve results compared to the N-BEATS interpretable model.

The results of the GRU general model have been analyzed for 7 days ahead of energy consumption prediction in terms of the MAPE, MAE, MSE and RMSE. In the “no covariates” scenario, the GRU achieved 56.76, 1.76, 8.26 and 2.87; the N-BEATS interpretable results have remained better than the smoothed data. On the other hand, the MAPE, MAE, MSE and RMSE for the “day as covariates” remained at 53.20, 2.08, 9.95 and 3.15, respectively. Suppose we compare these results with the “day as covariates” scenario, the N-BEATS interpretable with smoothed data and the GRU general model, the performance of the N-BEATS interpretable model in both scenarios of “no covariates” and “day as covariates” remained better. There is a significant improvement in the performance of the N-BEATS interpretable model compared to the GRU general with smoothed data.

If we compare the results of the TCN model in terms of MAPE, MAE, MSE and RMSE, with “no covariates,” it has remained at 68.61, 2.08, 10.06 and 3.17. For the 7 days ahead energy consumption prediction N-BEATS has outperformed TCN without smoothing and with smoothing data with a significant difference in the performance. The MAPE, MAE, MSE and RMSE for the “day as covariates” remained 63.55, 2.00, 9.93 and 3.15, respectively. Compared with the N-BEATS interpretable with smoothed data and TCN general model, the performance of the N-BEATS interpretable model in both scenarios of “no covariates” and “day as covariates” remained better.

The LSTM general model, MAPE, MAE, MSE and RMSE, with “no covariates”, have remained at 78.71, 2.35, 12.88 and 3.59. The “day as covariates” remained at 63.31, 2.27, 12.76 and 3.47. The N-BEATS interpretable with and without smoothing scenarios has outperformed the LSTM general model. If we compare these results with the “day as covariates” scenario of the N-BEATS interpretable without smoothed data, and the LSTM general model, the N-BEATS interpretable model remained dominant.

The GRU in terms of the MAPE, MAE, MSE and RMSE with “no covariates” scenario achieved 65.26, 2.12, 12.03 and 3.47; in comparison, the N-BEATS interpretable results have remained better compared without smoothing and smoothed data. On the other hand, the “day as covariates” results were 64.87, 2.81, 16.34 and 4.04, respectively. If we compare these results with the “day as covariates” scenario, the N-BEATS interpretable with smoothed data and the GRU general model, the performance of the N-BEATS interpretable model in both scenarios of “no covariates” and “day as covariates” remained better. There is a significant improvement in the performance of the N-BEATS interpretable model compared to the GRU general with smoothed data.

If we compare the results of the TCN model in terms of MAPE, MAE, MSE and RMSE, with “no covariates”, it has remained at 78.27, 2.37, 12.08 and 3.48. For the one-month-ahead energy consumption prediction, N-BEATS has outperformed TCN without smoothing and with smoothed data with a significant difference in performance. The MAPE, MAE, MSE and RMSE for the “day as covariates” remained 68.01, 2.19, 11.92 and 3.45, respectively. Compared with the N-BEATS interpretable with smoothed data and TCN general model, the performance of the N-BEATS interpretable model in both scenarios of “no covariates” and “day as covariates” remained better.

Figures 7 and 8 show the energy consumption of customer 789 for the second and third weeks of January 2013. It can be seen that the performance of N-BEATS has improved. The model has performed better than the other traditional deep learning models in terms of overall energy consumption.

The graph of the 3 weeks seems better compared to the two weeks of energy consumption. The performance of the models can be further improved by smoothening of the data, but the problem is disturbance of the original patterns of the energy consumption.

#### Month-ahead energy forecasting

We have considered four weeks of forecasting for the one month ahead, considering the previous pattern of weekly-based forecasting. As the number of days increases, the model’s performance decreases due to data fluctuations. The model’s performance for the month ahead scenario is lower than almost all scenarios. The proposed model has improved the performance in MAPE enhancement from 60.04 to 58.79 in the scenario of “no covariates.” The “day as covariates” has shown better results than the “no covariates,” as MAPE has improved from 66.46 to 55.49. The improvement is significant; the reason for the improvement is the proper training of the model to predict the patterns accurately. The MAE has been enhanced from 2.31 to 1.95 for the “no covariates,” and the “day as covariates” improved from 2.14 to 2.25. The model has achieved MSE with “no covariates” as 12.57, which has been enhanced to 8.22. The MSE of “day as covariates” has improved from 11.66 to 9.96. Similarly, the RMSE with “no covariates” has been enhanced from 3.54 to 2.87. The “day as covariates” RMSE can be noticed, improving it from 3.41 to 3.16.

The interpretable Blocked GRU, MAPE, MAE, MSE, and RMSE with “no covariates” scenario remained at 69.49, 2.15, 11.56, and 3.40. With the “day as covariates,” it has remained at 61.53, 2.35, 12.08, and 3.48. The interpretable Blocked LSTM showed identical results compared to the Blocked GRU. The results of the Blocked LSTM with “no covariates” scenario remained at 66.92, 2.11, 11.39, and 3.39. With the “day as covariates,” it has remained at 68.65, 2.18, 12.07, and 3.47. For the one-month (30 days) scenario, the N-BEATS interpretable model without and with smoothed data has outperformed the Blocked GRU and the Blocked LSTM in both “day as covariates” and “no covariates” scenarios.

The LSTM general model, MAPE, MAE, MSE and RMSE, with “no covariates”, have remained at 78.71, 2.35, 12.88 and 3.59. The “day as covariates” remained at 63.31, 2.27, 12.76 and 3.47. The N-BEATS interpretable with and without smoothing scenarios has outperformed the LSTM general model. If we compare these results with the “day as covariates” scenario of N-BEATS interpretable with LSTM general model using normal data the N-BEATS interpretable model remained dominant.

The results of the GRU in terms of the MAPE, MAE, MSE and RMSE with “no covariates” scenario have achieved 65.26, 2.12, 12.03 and 3.47; in comparison, the N-BEATS interpretable results have remained better compared with both without smoothing and smoothed data. On the other hand, the “day as covariates” results were 64.87, 2.81, 16.34 and 4.04, respectively. Suppose we compare these results with the “day as covariates” scenario, the N-BEATS interpretable with smoothed data and the GRU general model, the performance of the N-BEATS interpretable model in both scenarios of “no covariates” and “day as covariates” remained better. There is a significant improvement in the performance of the N-BEATS interpretable model compared to the GRU general with smoothed data.

If we compare the results of the TCN model with “no covariates”, it has remained at 78.27, 2.37, 12.08 and 3.48. For the one-month-ahead energy consumption prediction, the N-BEATS interpretable outperformed the TCN without smoothing and with smoothing data with a significant difference in performance. The MAPE, MAE, MSE and RMSE for the “day as covariates” remained 68.01, 2.19, 11.92 and 3.45, respectively. Compared with the N-BEATS interpretable with smoothed data and TCN general model, the performance of the N-BEATS interpretable model in both scenarios of “no covariates” and “day as covariates” remained better.

The specific performance of the models on the data of customer 789 can be seen in Fig. 9. The graphs show energy consumption for the four weeks. It can be seen that with the longer duration, the model’s performance has improved. This is further confirmed by Fig. 10 showing energy consumption for one year.

To summarize, the results of the one day ahead have remained better in terms of the MAPE, MAE, MSE and RMSE. However, the algorithms have struggled to tackle the complex time series data regarding training time and further evaluation. The general models’ training time remained lower than the interpretable models for the one-day ahead energy consumption. Different factors contributed to the results for the MAPE. The problem is that any “0” value in the actual energy consumption halts the calculation of MAPE. Further, the error remained higher due to the slight difference in actual and predicted values. The reason for the selection of day covariates was the daily data; hence it has boosted the performance of models all day, one, two, three and four weeks ahead of energy consumption prediction.

#### Statistical analysis and performance comparison with traditional models

The results of the models have been statistically analyzed for validation, as seen in Table  4. The standard deviation error of the N-BEATS interpretable was 0.07550, which is lower than the other models except for the LSTM interpretable. The reason for the same performance is the LSTM layers in N-BEATS, making them structurally the same, resulting in similar performance. The GRU algorithm also performed better; although it is lower than the N-BEATS, it outperforms the other algorithms. If we observe the standard deviation, N-BEATS has 1.44 while the LSTM interpretable has 1.38, with a variance of 1.910 and 208. These results are for the data of one customer for the year and hence may not be considered overall results, and every customer has a different standard deviation error.

**Table 4:** Statistical analysis of the prediction results.

Descriptive statistics							
	N Statistic	Minimum Statistic	Maximum Statistic	Mean Statistic	Std. error	Std. deviation Statistic	Variance Statistic
Actual	365	1.00	13.85	5.8590	0.15901	3.03783	9.228
BlockedGRU	365	2.42	10.61	5.9283	0.09325	1.78162	3.174
BlockedLSTM	365	3.32	9.95	6.2640	0.07233	1.38187	1.910
LSTM	365	2.29	10.56	6.4005	0.09633	1.84033	3.387
GRU	365	2.91	10.51	6.3136	0.08539	1.63132	2.661
TCN	365	1.55	10.63	6.3206	0.09627	1.83915	3.382
N-BEATS	365	2.60	9.55	5.6002	0.07550	1.44233	2.080

The performance of the model has been compared with the traditional deep learning models presented in Table  5. The comparison has been carried out based on MAE, as the traditional method has used the MAE parameter. While the traditional methods have used data having a smooth pattern, the proposed model has performed better than the traditional methods. It must be noted that the traditional method presented uses different datasets. Although we have applied the GRU on the same dataset used for this study, it can be seen in Table  3 that with the London households dataset, the performance of GRU is better compared with the^87^. The results have proved that the performance of deep learning algorithms depends entirely on the nature of the data. Although deep learning methods have a solid capability to learn complex patterns with accuracy, every algorithm has limitations. Also, the amount of data improves performance but increases the complexity and computation time. In terms of time, the N-BEATS can achieve better results than traditional algorithms in the minimum possible time frame. It can be seen that the deep learning algorithms have struggled with the data as the traditional deep learning methods DNN has shown an MAE of 23.5. While similarly, the Recurrent neural network has shown an MAE of 22.4. The gated recurrent unit (GRU) has shown an MAE of 22.5; if we compare the results with the proposed N-BEATS interpretable model, the MAE is 2.25 with the “day as covariates” and smoothed data. If we observe the detailed Table  3, the N-BEATS without covariates have also performed better. The proposed model’s higher MAPE value is due to the uncertainties of data and the energy consumption behavior of different customers. The proper mechanism of handling the uncertainty will improve the model’s performance. Different pre-processing approaches remain helpful to handle the uncertainty of data. The other main improvement in the MAPE can be achieved by applying the clustering technique to cluster customers with the same energy consumption patterns and then applying the prediction algorithm like N-BEATS. The interpretability of the proposed model is the main advantage and reason for the improvement in the error rate compared to the traditional deep learning methods.

**Table 5:** Comparative analysis.

Model	Forecast horizon	Metrics			
		MAPE	MAE	MSE	RMSE
DNN^87^	30 day ahead	-	23.5	-	–
RNN^87^	30 day ahead	-	22.4	-	–
GRU^87^	30 day ahead	-	22.5	-	
N-BEATS-interpretable	30 day ahead	55.49	2.25	9.96	3.16

## Conclusion

Various energy consumption prediction models have been proposed in the literature, but they face problems and fail to predict future energy consumption. There are various factors involved in the failure, but the most critical is the uncertainty of the data. Dealing correctly with the data’s uncertainty improves prediction algorithms’ performance. The proposed model handles the data uncertainty problem with data pre-processing to enhance the performance of the deep learning algorithms and provides a comparative analysis. The paper focuses on short-term (daily, weekly, and monthly) energy consumption forecasting using the N-BEATS interpretable method. The model’s first module is divided into two modules; the first is the smoothing of data, and the second is the prediction module incorporating the N-BEATS interpretable. The reason behind the success of the model is the N-BEATS interpretable algorithm which handles the time series data with accuracy. Further, deep learning has a robust problem-solving capability with big data without dividing the problem into sub-problems.

The detailed statistical comparative analysis of the N-BEATS with the LSTM general, LSTM interpretable, GRU general, GRU interpretable, and temporal convolutional network (TCN) has proved that the interpretable models have a strong capability of dealing with time series data. In contrast, the general models have been shown to be better than the N-BEATS interpretable models in training and running time. The performance of LSTM and GRU interpretable models has remained slightly lower than the N-BEATS interpretable. The general models have less complex input parameters without consideration of the covariates; hence, they show better performance in terms of training time.

Further, they do not observe the data patterns closely compared to the interpretable models. Further, the pre-processing module of the normalization of data significantly improved the results of N-BEATS compared to the normalization or smoothing of the data. However, the uncertainty of the data has remained challenging for the algorithms, specifically in terms of the MAPE. Hence, in future work, we will apply further pre-processing to smooth the data, but it might amend the original patterns of the energy consumption data. The covariates have increased the challenges of the algorithms, so we will consider the data of one year for each customer for the proper cycle, improving the model’s performance. The proposed study has used the trend scenario; the future study explores the models’ seasonal scenarios for a better comparative analysis.

## Data Availability

The data are available at London datastore (SmartMeter Energy Consumption Data in London Households) https://data.london.gov.uk/dataset/smartmeter-energy-use-data-in-London-households. The corresponding author may be contacted for further clarification regarding data.
